# Everyday discrimination alters cognitive benefits of restorative non-rapid eye movement sleep in Black adults

**DOI:** 10.1093/sleepadvances/zpag105

**Published:** 2026-09-24

**Authors:** Darlingtina K Esiaka, Sunkanmi Folorunsho, Stirling Crawford, Osswaah Fariduddin, Roland J Thorpe Jr., Elizabeth A Luth

**Affiliations:** Center for Health, Engagement and Transformation, University of Kentucky College of Medicine, Lexington, KY, United States; Department of Behavioral Science, University of Kentucky College of Medicine, Lexington, KY, United States; Sanders Brown Center on Aging, University of Kentucky, Lexington, KY, United States; Department of Sociology, University of Nebraska, Lincoln, NE, United States; Center for Health, Engagement and Transformation, University of Kentucky College of Medicine, Lexington, KY, United States; Department of Behavioral Science, University of Kentucky College of Medicine, Lexington, KY, United States; Center for Health, Engagement and Transformation, University of Kentucky College of Medicine, Lexington, KY, United States; Department of Molecular Biology, University of Kentucky, Lexington, KY, United States; Johns Hopkins Alzheimer's Disease Resource Center for Minority Aging Research, Johns Hopkins School of Public Health, Baltimore, MD, United States; Program for Research on Men's Health, Hopkins Center for Health Disparities Solutions, Johns Hopkins School of Public Health, Baltimore, MD, United States; Department of Family Medicine and Community Health, Robert Wood Johnson Medical School, Rutgers University, New Brunswick, NJ, United States

**Keywords:** cognition, home sleep monitoring, slow-wave sleep, NREM sleep

## Abstract

**Study Objectives:**

Restorative non-rapid eye movement sleep has been linked to cognitive functioning. Yet, it remains unclear whether experiences of discrimination, a persistent form of identity-based adversity, shape how objectively measured restorative non-rapid eye movement sleep relates to cognitive outcomes in Black Americans.

**Materials and Methods:**

Ninety-two Black adults completed the Everyday Discrimination Scale, a cognitive assessment, and three nights of at-home sleep monitoring. Restorative non-rapid eye movement sleep was indexed using N2–N3 delta power. Moderation analyses evaluated whether everyday discrimination altered the association between restorative non-rapid eye movement sleep and global cognition, assessed with the Montreal Cognitive Assessment, as well as visuospatial memory, assessed with the Benson Complex Figure task, adjusting for age, sex, marital status, education, subjective socioeconomic status, body mass index, and sleep efficiency.

**Results:**

Discrimination moderated the relationship between restorative non-rapid eye movement sleep and global cognition. At lower levels of discrimination, greater levels of restorative non-rapid eye movement sleep corresponded to higher global cognition scores, whereas at higher levels of discrimination, a greater level of restorative non-rapid eye movement sleep corresponded to lower global cognition scores. The interaction between restorative non-rapid eye movement sleep and discrimination on visuospatial memory was not significant after full covariate adjustment, with education emerging as a significant independent predictor of visuospatial memory.

**Conclusions:**

The findings suggest that associations between restorative non-rapid eye movement sleep and global cognition vary across levels of everyday discrimination among Black adults, independent of socioeconomic and health-related factors. These results highlight discrimination as a relevant contextual factor to consider when examining sleep-cognition relationships in cognitive aging.

## Teaser

This study demonstrates that everyday discrimination modifies association between restorative non-rapid eye movement sleep and global cognition in Black adults. The findings indicate that greater restorative non-rapid eye movement sleep activity is not uniformly linked to better cognitive performance and that social adversity may alter expected sleep-related benefits. By integrating objective sleep measures with lived experience, this work advances a more context-sensitive view of sleep and cognitive aging. Important gaps remain in identifying underlying physiological pathways and in clarifying directionality over time. Future studies should use longitudinal and mechanistic approaches and evaluate interventions that address both sleep and social conditions, with implications for reducing disparities in cognitive health.

## Graphical abstract

**Graphical abstract:**
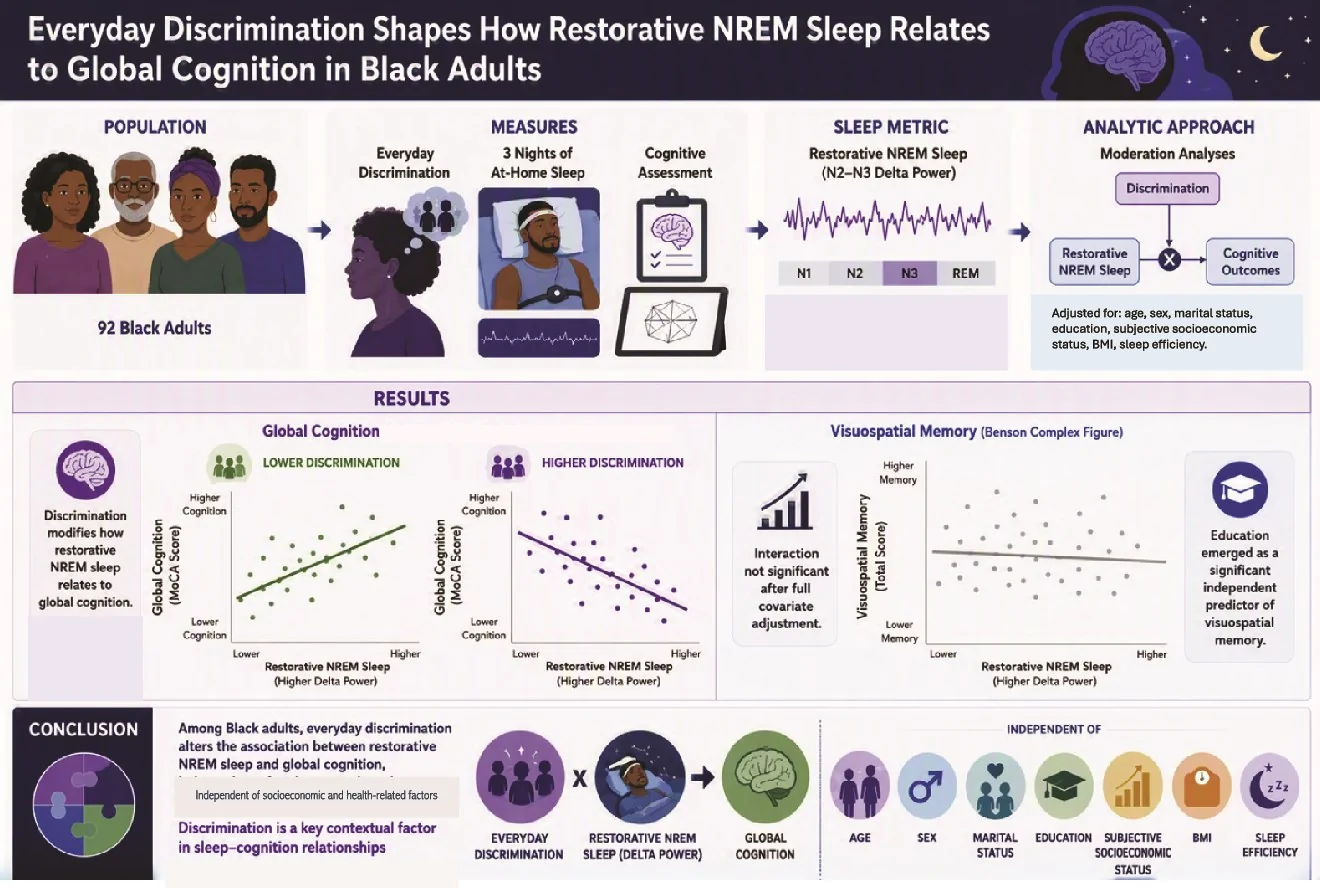

## Introduction

The age structure of the US population has shifted markedly toward older adulthood [1], a pattern expected to continue and one that heightens the public health importance of understanding risk and resilience in cognitive aging and dementia [2, 3]. Black Americans experience disproportionately higher rates of adverse cognitive aging outcomes, including Alzheimer’s Disease and related dementias (ADRD), reflecting persistent disparities documented in the literature [4, 5]. The disparities in cognitive aging and ADRD risk have been linked to factors such as structural inequities, cumulative life-course disadvantage, and longstanding differences in social and environmental conditions [6–8]. Against the backdrop of these enduring social and structural disparities, sleep has emerged as a salient health-related factor that may be relevant to understanding variation in cognitive outcomes across adulthood.

Sleep is an important modifiable factor for cognitive health in later life [9–12]. Reductions in deep sleep have been associated with poorer memory, greater cognitive decline, and accelerated brain aging [13–15]. In particular, slow-wave sleep, the deepest stage of non-rapid eye movement (NREM) sleep, supports processes relevant to cognitive health, including memory consolidation, synaptic regulation, and restoration of neural systems engaged during wakefulness [16]. Delta-frequency activity during NREM sleep reflects synchronized cortical activity associated with sleep depth and homeostatic sleep pressure. Lower slow-wave activity has been associated with poorer memory, reduced brain integrity, cognitive decline, and greater ADRD pathology, suggesting that NREM delta power may be relevant to cognitive-aging vulnerability [17–19].

Chronic psychosocial stress may modify association between NREM slow-wave activity and cognition. Everyday discrimination is characterized by recurrent, subtle, and routine experiences of unfair treatment that can operate as a chronic psychosocial stressor [20]. As a persistent form of identity-based adversity, discrimination has been linked to poorer cognitive outcomes and heightened vulnerability to adverse cognitive aging trajectories among Black adults [6, 21, 22]. One framework for understanding these associations is allostatic load, which conceptualizes chronic stress as the cumulative physiological impact resulting from repeated demands on adaptive systems over time [23, 24]. Because experiences of discrimination often occur repeatedly across everyday contexts, they may contribute to sustained vigilance, anticipatory stress, and prolonged activation of stress-response systems across the life course [25–28].

Experiences of everyday racial discrimination have also been associated with reduced sleep duration and greater sleep disturbance, even after accounting for socioeconomic status and other common stressors [29–33]. Despite growing interest in cognitive aging disparities, limited research has examined how objectively measured sleep physiology interacts with experiences of discrimination to shape cognitive outcomes among Black Americans. Moreover, existing studies have largely focused on older adult samples, with relatively little attention to middle-aged Black adults, a critical period for understanding the emergence and accumulation of life-course risk trajectories relevant to later-life cognitive aging.

Preliminary evidence has begun to suggest that experiences of discrimination may interact with slow-wave sleep to shape memory performance among aging Black adults, highlighting a critical intersection between psychosocial stress and sleep physiology [34]. The current study examined within-group variation in experiences of discrimination among Black adults and tested whether the experiences of discrimination moderated the association between objectively measured sleep and cognitive outcomes. We focused on electroencephalographic (EEG) delta power during NREM sleep stages N2 and N3, which was used as an indicator of sleep depth and restorative function. Our primary hypothesis was that the association between restorative NREM sleep (indexed by EEG delta power during NREM stages N2 and N3) and global cognition would vary according to the level of discrimination.

Global cognition was selected as the primary outcome because chronic stress and sleep physiology may have consequences across multiple cognitive systems. The Montreal Cognitive Assessment measures attention, executive function, memory, language, conceptual thinking, calculation, and orientation and can detect subtle multidomain variation in adults without clinically evident impairment [35]. Its multidomain structure is particularly relevant to this middle-aged and older sample because the consequences of accumulated social adversity may emerge as modest differences across several cognitive processes before becoming evident as a pronounced deficit in one domain. Visuospatial memory was examined as an exploratory, domain-specific outcome because memory consolidation is one of the cognitive processes most consistently linked to NREM sleep [36–39]. Delayed reproduction of a complex figure requires the encoding, retention, organization, and retrieval of nonverbal information. Visuospatial memory may therefore provide a more targeted test of whether the hypothesized interaction was evident. In addition, impairments in visuospatial memory have been identified as possible early indicators of subsequent cognitive decline and dementia risk. Examining both global cognition and visuospatial memory allowed us to determine whether the interaction was broadly evident across global cognitive functioning or was also detectable in a theoretically relevant, domain-specific measure.

## Materials and methods

### Design and participants

This study used a cross-sectional observational design to examine whether everyday discrimination moderates the association between objectively measured restorative NREM sleep and cognitive functioning among Black adults. The participants were self-identified Black or African American men and women, living in the Lexington and Louisville areas of Kentucky. The participants were 40 years of age or older and able to understand English. They were recruited through the University of Kentucky Center for Clinical and Translational Science Participant Recruitment Services, as well as through targeted flyer distributions at Black-owned businesses and community locations. After providing informed consent, participants completed the measures described below and underwent a cognitive assessment administered according to standardized protocol. Participants were then instructed on the use of the Sleep Profiler device and completed three consecutive nights of at-home sleep monitoring. Sleep recordings were processed using automated staging procedures to derive NREM stage N2–N3 delta power and sleep efficiency measures. Trained research personnel administered the cognitive assessments during scheduled study visits after sleep monitoring. However, the time of day at which cognitive testing occurred was not standardized across participants, as assessments were conducted based on participants' availability. Consequently, the present analyses focused on the mean NREM sleep obtained across three nights of at-home monitoring rather than on the acute effects of sleep on next-day cognitive performance. All study procedures were reviewed and approved by the University of Kentucky Institutional Review Board (#90016). All participants were compensated for study completion.

### Measures

#### Outcome variables: cognitive function

The primary outcome variable, global cognition, was measured using the Montreal Cognitive Assessment (MoCA) [35], a widely used, validated, and sensitive multidomain measure appropriate for detecting subtle cognitive variation in aging populations. Compared to other global screening tools, it demonstrates superior sensitivity to early-stage decline, making it appropriate for a midlife-to-older adult sample where deficits may be subclinical. The MoCA assesses multiple cognitive domains, including attention and concentration, executive function, memory, language, conceptual thinking, calculations, and orientation. It has been widely used in studies of racial/ethnic health disparities and aging, with evidence supporting its utility in Black American samples when interpreted with appropriate contextual considerations such as education and sociocultural factors [40, 41]. Scores range from 0 to 30, with higher scores indicating better cognitive performance; a score of 26 or above is generally considered indicative of normal cognitive function. Although scores of 26 or higher are commonly considered within the normal range, MoCA scores were analyzed continuously to capture multidomain cognitive variation rather than classify impairment. Because performance can vary with educational, cultural, and demographic factors, scores below 26 were not interpreted as evidence of clinical impairment.

The exploratory outcome, visuospatial memory, was measured using the Benson Complex Figure Delayed Recall [42], a standardized neuropsychological measure of nonverbal memory. In this task, participants were first asked to copy a complex geometric figure and, following a delay period, to reproduce the figure from memory. The delayed recall condition specifically assesses visuospatial memory by evaluating the accuracy and organization of the reproduced figure. Scores range from 1 to 16, with higher scores indicating better visuospatial memory function.

#### Predictor variable: restorative NREM sleep

Restorative NREM sleep was assessed using an at-home polysomnography approach with the Sleep Profiler (Advanced Brain Monitoring, Carlsbad, CA). The Sleep Profiler is a wireless, self-applied device that records EEG, electrooculographic, and electromyographic signals. Continuous recordings were obtained across three consecutive nights to account for night-to-night variability, and primary sleep metrics were averaged across nights for analysis. The device recorded three frontopolar differential EEG signals using electrode locations AF7, AF8, and Fpz, yielding the AF7–AF8, AF7–Fpz, and AF8–Fpz derivations. EEG signals were sampled at 256 Hz. The recorded signals were subsequently processed using the manufacturer’s signal-processing procedures, including filtering and artifact rejection. Sleep staging algorithms classified sleep into standard stages, including NREM stages N2 and N3. Delta power was defined by the Sleep Profiler analysis software as EEG spectral delta power within the 0.5–4.0 Hz frequency band during NREM stages N2 and N3. Delta power reflects the magnitude of low-frequency EEG activity during NREM sleep and was used as an index of restorative NREM sleep physiology. Spectral power values were obtained directly from the Sleep Profiler output and analyzed as provided by the manufacturer’s software. Values were averaged across the three nights of monitoring to provide a measure of habitual NREM delta activity. Sleep efficiency, the percentage of time in bed actually spent asleep, was also obtained from the automated staging outputs and included as a covariate.

#### Moderator variable: everyday discrimination

The Everyday Discrimination Scale [20] was assessed using a nine-item, self-report instrument that measures the frequency of routine, subtle, and daily experiences of unfair treatment. Participants were asked, “In your day-to-day life, how often have any of the following things happened to you?” Examples include, “people treat you with less respect” and “people act as if they’re afraid of you.” Response choices range from “never” to “almost every day.” The scale yielded a reliability score of Cronbach’s α = .89 in this study.

#### Covariates

Demographic and health-related variables were included as covariates to account for potential confounding influences on sleep architecture, neurophysiological measures, and cognitive outcomes. Age, sex, and marital status were incorporated given their established associations with sleep patterns and cognitive aging [43–45]. Education was included given its well-documented associations with cognitive reserve and neurocognitive outcomes across adulthood [46]. Subjective socioeconomic status (SES), assessed using the MacArthur Ladder (a validated 1–10 scale of perceived social standing), was included as a proxy for broader socioeconomic position, given evidence linking financial hardship and social disadvantage to both sleep health and cognitive aging [8, 27, 33]. Body mass index (BMI) was included because obesity-related metabolic factors have been associated with sleep architecture and cognitive performance [13, 47]. Sleep efficiency, derived from the Sleep Profiler automated staging outputs, was included to distinguish the specific contribution of slow-wave delta power from overall sleep continuity and quality. Inclusion of these variables as covariates was intended to improve model precision and reduce bias in estimating the primary associations of interest.

### Data analysis

Descriptive statistics and bivariate Pearson correlations were computed to characterize the sample and examine associations among study variables prior to hypothesis testing. Internal consistency for the Everyday Discrimination Scale was assessed using Cronbach’s alpha.

To examine whether everyday discrimination moderates the relationship between restorative NREM sleep and cognitive outcomes, moderation regression analyses were conducted following Hayes [48]. Restorative NREM sleep (N2–N3 delta power), averaged across three consecutive nights of at-home polysomnography, served as the predictor (X), and everyday discrimination served as the moderator (W). Both were mean-centered prior to forming the interaction term to reduce multicollinearity and facilitate the interpretation of main effects [48, 49]. All models were adjusted for age, sex, marital status, education, subjective SES, BMI, and sleep efficiency. The interaction term was entered in the final step of a hierarchical regression, following entry of the main effects and covariates in the preceding step. Model fit was evaluated using *R*^2^, adjusted *R*^2^, and the overall *F* statistic, and results are reported in the Results section.

To decompose significant interactions, simple slopes were examined at low (−1 *SD* below the mean), mean, and high (+1 *SD* above the mean) levels of everyday discrimination, and these conditional effects are reported in the Results section. Figure 1 provides a visual representation of the interaction pattern across both outcomes, with Panel A displaying the primary outcome and Panel B displaying the exploratory outcome. Residual normality was assessed for each model using the Shapiro–Wilk test. The analytic sample was *N* = 92 for the primary model and *N* = 89 for the exploratory model, after excluding participants with missing data on any analysis variable. A total of 96 participants were recruited into the ongoing study. For the primary analytic sample, four participants were excluded due to either missing with objective sleep data or everyday discrimination scores. For the exploratory Benson sample, seven were excluded for missing visuospatial memory scores, yielding *N* = 89. Statistical significance was set at α = .05, with marginal significance noted at α = .10. All analyses were conducted using Stata (Version 18.0; StataCorp, College Station, TX).

**Figure 1:**
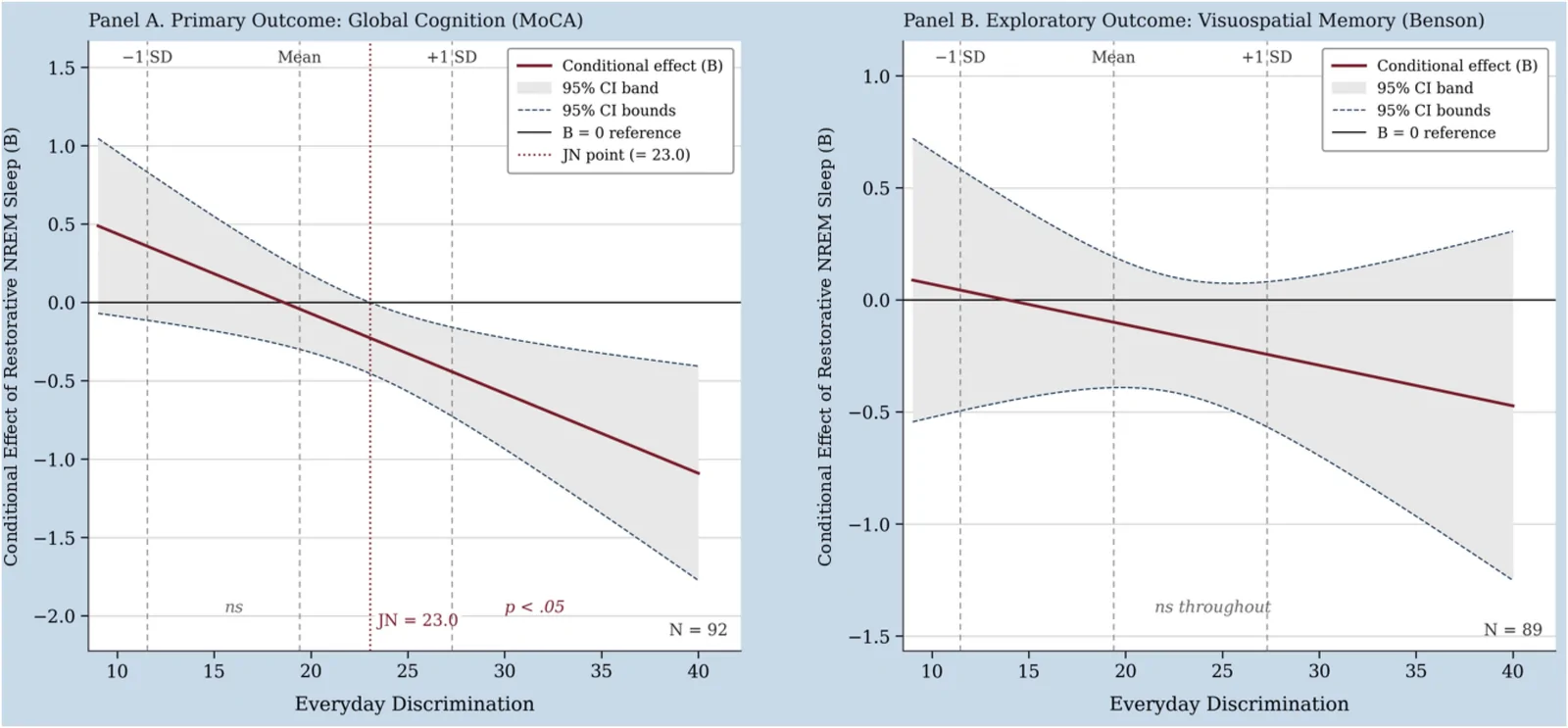
Moderating role of everyday discrimination in the association between restorative NREM sleep and cognitive outcomes in aging Black adults. Note. Covariates (age, sex, marital status, education, SES, BMI, sleep efficiency) held at sample means. Restorative NREM sleep and discrimination are mean-centered. Panel A displays the primary outcome (global cognition; *N* = 92), for which the restorative NREM sleep × discrimination interaction was characterized by a significant negative association between restorative NREM sleep and global cognition at high levels of discrimination. Panel B displays the exploratory outcome (visuospatial memory; *N* = 89), for which no significant interaction was observed despite a visually similar pattern. Restorative NREM sleep reflects slow-wave EEG delta power during NREM stages 2–3, averaged across three nights of at-home polysomnography.

## Results

### Sample characteristics

The characteristics of the analytic sample that consisted of 92 Black and African American adults are displayed in Table 1. Participants ranged in age from 40 to 82 years (*M* = 53.47, *SD* = 8.47). The majority were females (*n* = 61, 66.3 per cent), with 33.7 per cent identifying as male (*n* = 31). Most were US-born (*n* = 67, 72.8 per cent), while 27.2 per cent were foreign-born (*n* = 25). The sample was highly educated, with 57.6 per cent holding a graduate degree (*n* = 53), followed by a college degree (26.1 per cent, *n* = 24), high school diploma or General Educational Development (GED; 14.1 per cent, *n* = 13), and some college (2.2 per cent, *n* = 2). The largest marital status groups were married or partnered (32.6 per cent, *n* = 30), cohabitating (30.4 per cent, *n* = 28), and widowed (25.0 per cent, *n* = 23). Participants’ mean SES was 5.73 (*SD* = 1.71), and their mean BMI was 32.69 (*SD* = 8.28).

**Table 1:** Demographic and clinical characteristics of 92 Black and African American adults

Variable	*n*	*M* (*SD*) or %
Age (years)		53.47 (8.47)
Range	40–82	
Sex		
Female	61	66.3
Male	31	33.7
Nativity		
US-born	67	72.8
Foreign-born	25	27.2
Education		
High school/GED	13	14.1
Some college	2	2.2
College degree	24	26.1
Graduate degree	53	57.6
Marital status		
Married/partnered	30	32.6
Separated	1	1.1
Divorced	3	3.3
Widowed	23	25.0
Never married	7	7.6
Cohabitating	28	30.4
SES (range 1–10)		5.73 (1.71)
BMI		32.69 (8.28)
Global cognition (primary outcome; range 13–30)		23.08 (3.30)
Visuospatial memory (exploratory outcome; range 1–16)		10.12 (3.67)
Everyday discrimination (range 9–40)		19.40 (7.87)
Restorative NREM sleep (N2–N3 delta power; avg across three nights)		4.610 (2.881)
Sleep efficiency (%)		71.86 (12.34)

*N* = 92. Restorative NREM sleep was indexed by EEG delta power at 0.5–4.0 Hz during N2 and N3, averaged across three nights of at-home Sleep Profiler monitoring.

Global cognition scores ranged from 13 to 30 (*M* = 23.08, *SD* = 3.30). Visuospatial memory scores ranged from 1 to 16 (*M* = 10.12, *SD* = 3.67). Participants reported moderate levels of everyday discrimination (*M* = 19.40, *SD* = 7.87, range = 9–40); the Everyday Discrimination Scale demonstrated excellent internal consistency (α = .891). Restorative NREM sleep (i.e. N2–N3 delta power) averaged 4.610 (*SD* = 2.881) across 3 nights of at-home polysomnography, with an average sleep efficiency of 71.9 per cent (*SD* = 12.34).

### Bivariate correlations

Bivariate correlations among study variables are shown in Table 2. Marital status was significantly and negatively correlated with global cognition (*r* = −.26, *p* < .05), indicating that participants who were widowed or unmarried tended to have lower global cognition scores. Age was marginally and negatively associated with global cognitive function (*r* = −.20, *p* < .10). Sex was significantly correlated with restorative NREM sleep (*r* = −.21, *p* < .05), with males showing lower slow-wave activity. Education was significantly and positively correlated with global cognition (*r* = .31, *p* < .01), indicating that higher educational attainment was associated with better global cognitive performance. Everyday discrimination and restorative NREM sleep were not significantly correlated (*r* = .06, *p* > .05), ruling out multicollinearity between the predictor and moderator.

**Table 2:** Association among cognition, restorative NREM sleep and study covariates

Variable	1	2	3	4	5	6	7	8	9	10
1. Global cognition	—	—	—	—	—	—	—	—	—	—
2. Restorative NREM sleep	−0.14	—	—	—	—	—	—	—	—	—
3. Discrimination	−0.09	0.06	—	—	—	—	—	—	—	—
4. Age	−0.20^†^	−0.16	0.00	—	—	—	—	—	—	—
5. Sex (M = 1)	−0.12	−0.21^*^	−0.02	0.13	—	—	—	—	—	—
6. Marital status	−0.26^*^	0.08	0.08	0.15	−0.16	—	—	—	—	—
7. Education	0.31^**^	0.14	0.17	−0.30^**^	−0.16	−0.24^*^	—	—	—	—
8. SES	0.00	0.01	−0.06	−0.03	−0.05	0.00	0.19^†^	—	—	—
9. BMI	−0.08	0.04	0.19^†^	−0.11	−0.13	0.06	−0.11	0.02	—	—
10. Sleep efficiency	0.20^†^	−0.10	0.15	−0.27^**^	0.12	−0.02	0.38^**^	0.01	−0.04	—
***M***	23.08	4.61	19.40	53.47	0.34	3.65	3.27	5.73	32.69	71.86
***SD***	3.30	2.88	7.87	8.47	0.48	2.06	1.05	1.71	8.28	12.34

*N* = 92. Values below the diagonal are Pearson correlation coefficients.

^†^ *p* < .10. ^*^p < .05. ^**^p < .01.

### Moderation analysis

The results of the moderation analysis are presented in Table 3. To examine whether everyday discrimination moderates the relationship between restorative NREM sleep and cognitive outcomes, moderation regression analyses were conducted following Hayes^48^ Restorative NREM sleep served as the predictor variable and everyday discrimination as the moderator variable, with age, sex, marital status, education, SES, BMI, and sleep efficiency included as covariates. Both the predictor and moderator variables were mean-centered prior to forming the interaction term. Residual normality was confirmed for both models (Global cognition: Shapiro–Wilk W = 0.9896, *p* = .6883; Visuospatial memory: W = 0.9869, *p* = .5145).

**Table 3:** Moderation regression predicting cognitive outcomes: restorative NREM sleep × everyday discrimination

Variable	*B*	*SE*	*t*	*P*	95% CI
**Global cognition [primary outcome]** *R*^2^ = 0.2828, Adj *R*^2^ *=* 0.1942, *F*(10, 81) *=* 3.193, *p* = .0017					
Intercept	21.5807	2.7615	7.815	< .001^***^	16.086 to 27.075
Restorative NREM sleep (X)	−0.0414	0.1294	−0.320	.7497	−0.299 to 0.216
Discrimination (W)	−0.0555	0.0420	−1.322	.1899	−0.139 to 0.028
Restorative NREM sleep × discrimination	−0.0509	0.0187	−2.719	.0080^**^	−0.088 to −0.014
Age	−0.0432	0.0410	−1.053	.2954	[−0.125, 0.038]
Sex (male = 1)	−1.1284	0.7128	−1.583	.1173	−2.547 to 0.290
Marital status	−0.3081	0.1637	−1.882	.0634^†^	−0.634 to 0.018
Education	0.4065	0.3863	1.052	.2959	−0.362 to 1.175
SES	0.0395	0.1963	0.201	.8410	−0.351 to 0.430
BMI	−0.0301	0.0400	−0.751	.4546	−0.110 to 0.050
Sleep efficiency	0.0347	0.0293	1.186	.2393	−0.024 to 0.093
**Visuospatial memory [exploratory outcome]** *R*^2^ *=* 0.2634, Adj *R*^2^ = 0.1690, *F*(10, 78) = 2.789, *p =* .0053					
Intercept	5.3684	3.1295	1.715	.0902^†^	−0.862 to 11.599
Restorative NREM sleep (X)	−0.0996	0.1466	−0.679	.4989	−0.392 to 0.192
Discrimination (W)	−0.0404	0.0481	−0.839	.4041	−0.136 to 0.055
Restorative NREM sleep × discrimination	−0.0181	0.0213	−0.850	.3977	−0.060 to 0.024
Age	0.0520	0.0469	1.109	.2708	−0.041 to 0.145
Sex (male = 1)	−1.4168	0.8179	−1.732	.0872^†^	−3.045 to 0.211
Marital status	−0.0336	0.1859	−0.181	.8572	−0.404 to 0.336
Education	1.4975	0.4441	3.372	.0012^**^	0.613 to2.382
SES	−0.3927	0.2288	−1.716	.0901^†^	−0.848 to 0.063
BMI	0.0918	0.0461	1.993	.0498^*^	0.000 to 0.184
Sleep efficiency	−0.0047	0.0334	−0.140	.8892	−0.071 to 0.062

Primary outcome: global cognition (*N* = 92); exploratory outcome: visuospatial memory (*N* = 89). All models were adjusted for age, sex, marital status, education, subjective SES, BMI, and sleep efficiency. Restorative NREM sleep (X) and discrimination (W) are mean-centered prior to computing the interaction term. Residual normality—global cognition: Shapiro–Wilk W = 0.9896, *p* = .6883; visuospatial memory: W = 0.9869, *P* = .5145. ^†^*p* < .10. ^*^p < .05.

^**^ *p* < .01. ^***^p < .001.

#### Primary outcome: global cognition

The moderation model predicting global cognition was statistically significant (*R*^2^ = 0.2828, Adj *R*^2^ = 0.1942, *F*(10, 81) = 3.193, *p* = .0017), explaining approximately 28 per cent of the variance in global cognitive functioning. The interaction between restorative NREM sleep and everyday discrimination was statistically significant (*B* = −0.0509, *SE* = 0.0187, *t* = −2.719, *p* = .0080, 95% CI = −0.088 to −0.014), indicating that the cognitive benefit of slow-wave sleep activity on global cognition depends on the level of discrimination experienced, independent of education, SES, BMI, and sleep efficiency. Marital status showed a marginal negative association with global cognition (*B* = −0.3081, *SE* = 0.1637, *p* = .0634). None of the newly added covariates reached statistical significance in the primary model, confirming that the discrimination–sleep interaction on global cognition is not accounted for by socioeconomic or health-related factors.

The decomposition of the interaction is displayed in Table 4 and Figure 1. Simple slopes were examined at low (−1 *SD*), mean, and high (+1 *SD*) levels of discrimination. At low discrimination (score = 11.54), restorative NREM sleep was positively but not significantly associated with global cognition (*B* = 0.3592, *SE* = 0.2379, *t* = 1.510, *p* = .1349, 95% CI = −0.114 to 0.833). At mean levels of discrimination, no significant association emerged (*B* = −0.0414, *SE* = 0.1294, *p* = .7497). Critically, at high discrimination (score = 27.27), restorative NREM sleep was significantly and negatively associated with global cognition (*B* = −0.4421, *SE* = 0.1426, *t* = −3.099, *p* = .0027, 95% CI = −0.726 to −0.158). These findings indicate that the association between restorative NREM sleep and global cognition shifted from positive at lower levels of discrimination to significantly negative at higher levels of discrimination.

**Table 4:** Conditional effects of restorative NREM sleep on cognitive outcomes at low, mean, and high levels of everyday discrimination

Level of discrimination	Score	*B*	*SE*	*t*	*P*	95% CI
***Panel A: Effect of restorative NREM sleep on global cognition at levels of discrimination (primary outcome, n = 92)***						
−1 *SD*	11.54	0.3592	0.2379	1.510	.1349	−0.114 to 0.833
Mean	19.40	−0.0414	0.1294	−0.320	.7497	−0.299 to 0.216
+1 *SD*	27.27	−0.4421	0.1426	−3.099	.0027^*^	−0.726 to −0.158
***Panel B: Effect of restorative NREM sleep on visuospatial memory at levels of discrimination (exploratory outcome, N = 89)***						
−1 *SD*	11.45	0.0437	0.2708	0.161	.8722	−0.495 to 0.583
Mean	19.37	−0.0996	0.1466	−0.679	.4989	−0.392 to 0.192
+1 *SD*	27.29	−0.2430	0.1627	−1.493	.1394	−0.567 to 0.081

Simple slopes represent the conditional effect of restorative NREM sleep on the cognitive outcome at −1 *SD*, mean, and +1 *SD* of everyday discrimination. All models adjusted for age, sex, marital status, education, subjective SES, BMI, and sleep efficiency. Panel A *df* = 81; Panel *B df* = 78.

^*^ *p* < .01.

#### Exploratory outcome: visuospatial memory

As an exploratory analysis, the moderation model was estimated with visuospatial memory as the outcome (*N* = 89), a domain sensitive to early cognitive decline. The overall model was statistically significant (*R*^2^ = 0.2634, Adj *R*^2^ = 0.1690, *F*(10, 78) = 2.789, *p* = .0053), explaining approximately 26 per cent of the variance. However, the restorative NREM sleep × discrimination interaction was not statistically significant (*B* = −0.0181, *SE* = 0.0213, *t* = −0.850, *p* = .3977, 95% CI = −0.060 to 0.024). The attenuation of this interaction following covariate adjustment indicates that the original visuospatial memory finding was partially explained by socioeconomic and health-related factors not included in the initial model. Notably, education emerged as a significant and positive predictor of visuospatial memory (*B* = 1.4975, *SE* = 0.4441, *p* = .0012), and BMI was also a significant predictor (*B* = 0.0918, *SE* = 0.0461, *p* = .0498). These findings suggest that the association between the discrimination–sleep interaction and visuospatial memory is largely accounted for by educational attainment and BMI.

## Discussion

In this study, we examined whether self-reported everyday discrimination moderated the relationship between restorative NREM sleep and cognitive performance in a community-based sample of middle-aged and older Black American adults, while adjusting for sex, age, marital status, education, socioeconomic status, BMI, and sleep efficiency. The findings are broadly in line with our hypothesized patterns. Everyday discrimination moderated the association between restorative NREM sleep and global cognition. The analysis was robust to full covariate adjustment, indicating that the interaction is not explained by socioeconomic or health-related confounding. At lower levels of discrimination, a higher level of restorative NREM sleep corresponded to more favorable global cognitive scores, whereas at higher levels of discrimination, higher levels of restorative NREM sleep corresponded to lower global cognitive scores.

Our findings indicate that associations between restorative sleep and global cognitive functioning may be contingent on psychosocial context. Under conditions of lower reported discrimination, the observed pattern is consistent with prior evidence linking greater slow-wave activity to more favorable cognitive outcomes in aging populations, particularly for domains relevant to memory consolidation and cognitive maintenance [50, 51]. In contrast, as exposure to everyday discrimination increases, the association between restorative NREM Sleep and cognition appears to attenuate and, at higher levels, may shift in an unfavorable direction. This pattern is broadly consistent with emerging work indicating that the cognitive relevance of sleep physiology is not uniform across individuals but may depend on social experiences that alter stress impacts and physiological regulation [52, 53]. These findings underscore the importance of incorporating psychosocial context when interpreting sleep–cognition associations in middle-to-later life, particularly in populations disproportionately exposed to chronic social stressors.

An important consideration is that the observed interaction does not indicate that restorative NREM sleep is inherently associated with poorer cognitive functioning. Rather, the findings suggest that the association between NREM delta activity and cognition may differ across levels of chronic psychosocial stress exposure. Although greater slow-wave activity is generally associated with more favorable cognitive outcomes, the present study did not observe a statistically significant positive association at lower levels of discrimination, and the cross-sectional design does not permit conclusions regarding the direction of these relationships. It is therefore possible that the observed interaction reflects differences in the cognitive significance of NREM delta activity across psychosocial contexts rather than differences in sleep physiology itself.

Several explanations may account for this unexpected pattern. Chronic exposure to discrimination-related stress may alter physiological processes that interact with sleep and cognition, such that greater NREM delta activity does not confer the same cognitive correlates under conditions of elevated stress impacts. Alternatively, the observed association may reflect unmeasured factors related to stress, health, or cognitive functioning that were not captured in the present study. It is also possible that global cognition, as assessed by the MoCA, may be less sensitive to sleep-related variation than specific cognitive domains such as attention, executive functioning, or memory consolidation, which have been more directly linked to slow-wave sleep in prior research. Future longitudinal studies incorporating repeated cognitive assessments and more domain-specific cognitive measures will be important for clarifying the mechanisms underlying these associations and determining whether the observed pattern persists over time.

Our exploratory analysis indicated that the discrimination × sleep interaction was not statistically significant in the fully adjusted visuospatial memory model. In contrast, educational attainment emerged as a robust independent correlate of visuospatial memory performance. This pattern suggests that variability in visuospatial memory within this sample is more closely aligned with educational differences than with the combined pattern of restorative NREM sleep and discrimination. Interpreted in context, educational attainment may reflect aspects of cognitive reserve that are relevant to performance in this domain [46], although this interpretation should be considered cautiously.

The absence of a significant interaction for visuospatial memory also has important implications for the interpretation of our broader theoretical framework. One possible interpretation is that the observed moderation effect does not extend uniformly across cognitive domains and may be more relevant to multidomain cognitive functioning than to domain-specific visuospatial memory performance. While we selected visuospatial memory because memory processes are often linked to NREM sleep, the present findings do not support the conclusion that discrimination consistently modifies associations between restorative NREM sleep and all cognitive outcomes. Instead, the results suggest potential domain-specific differences in how sleep physiology and psychosocial stress relate to cognitive functioning. Whereas global cognition showed evidence of a discrimination-dependent association with restorative NREM sleep, visuospatial memory did not exhibit a comparable pattern after adjustment for covariates. This divergence highlights the need for future studies to examine a broader range of cognitive domains, particularly attention, executive functioning, and memory processes that may be more directly sensitive to sleep-related variation.

While not the primary focus of the current study, we also observed sex-related differences across sleep-related physiology, with males demonstrating lower restorative NREM sleep activity. These findings align with prior work documenting sex differences in sleep architecture, including NREM EEG characteristics, as well as sex-related variation in cognitive performance across aging cohorts [43, 45]. The absence of significant associations for sleep efficiency in both models suggests that the observed interaction is not simply attributable to general sleep quality as captured by sleep efficiency, but rather to the specific operationalization of restorative NREM sleep. The current results therefore add to the literature on the importance of accounting for demographic heterogeneity when examining sleep–cognition associations in diverse aging populations.

This study has limitations that should be considered. The cross-sectional design prevents the determination of temporal ordering and does not support the conclusion that greater NREM delta activity causes poorer cognition under high discrimination. Although the sample size was sufficient to detect the primary interaction effect, it limited the ability to conduct advanced stratified or subgroup analyses such as analyses of sex differences. The sample was also highly educated, which may constrain generalizability to broader populations with greater socioeconomic variability. We captured subjective SES rather than objective socioeconomic indicators; future work should include income, wealth, and neighborhood-level indicators. Future longitudinal studies with larger and more socioeconomically diverse samples are needed to evaluate the stability of these associations over time and to determine their relevance for clinically meaningful cognitive decline. Cognitive testing time was not standardized, and sleep immediately preceding testing was not evaluated. Mental and psychological health were also not assessed, although these factors may relate to perceived discrimination, sleep, and cognition. Additionally, because the study examined within-group variation among Black adults without a racial or ethnic comparison group, the observed pattern should not be interpreted as unique to Black adults.

Furthermore, measurement limitations warrant consideration. N2–N3 delta power provides an objective indicator of restorative sleep but lacks standardized clinical cut points; discrimination was assessed via self-report without attribution or temporal framing; and cognitive measures may reflect educational and sociocultural influences. These features may shape the interpretability and generalizability of the findings. An additional consideration is the operationalization of restorative sleep using N2–N3 delta power. Although this measure captures slow-wave activity across deeper stages of NREM sleep, it does not distinguish the relative contribution of N2 versus N3 sleep. Future studies should examine whether similar associations are observed using N3 sleep specifically, which is generally considered the deepest stage of NREM sleep. In addition, other indicators of sleep continuity and fragmentation, including wake after sleep onset, early morning awakening, and related measures, may provide complementary information regarding potential pathways linking discrimination, sleep physiology, and cognitive functioning.

Despite the aforementioned limitations, our findings have several implications for research on cognitive aging and sleep. First, they suggest that sleep-related physiological markers should not be interpreted in isolation from their social and environmental context, as their associations with cognitive outcomes may vary meaningfully across different lived experiences. This is particularly relevant for studies seeking to identify sleep-based indicators of cognitive health or risk, as the utility of such markers may depend on exposure to chronic psychosocial stressors. Second, the results underscore the importance of integrating measures of discrimination and other adverse social exposures into models of cognitive aging, as omission of these factors may obscure heterogeneity in sleep–cognition relationships. From a clinical and public health perspective, the findings further highlight the need for a broader approach to cognitive aging research that extends beyond biological indicators alone and incorporates social determinants that may shape cognitive trajectories over time.

## Conclusion

We examined the relationship between sleep and cognitive outcomes and found that associations between restorative NREM sleep and cognitive outcomes are not consistent across individuals and may differ depending on psychosocial context. The observed pattern is consistent with a broader literature emphasizing that sleep–cognition relationships vary according to social and environmental conditions. The results highlight the importance of considering variation in exposure to chronic psychosocial stress, such as the experiences of discrimination, when interpreting sleep-based indicators of cognitive aging. More broadly, these findings support the view that integrating social context into models of sleep and cognition may improve understanding of heterogeneity in cognitive trajectories and risk for later-life cognitive impairment. Thus, the current study adds to a growing body of literature emphasizing the importance of integrating social experience with biological measures in studies of cognitive aging and dementia-related risk.

## Data Availability

The data analyzed in the current paper are from an ongoing study and are available from the corresponding author upon reasonable request.
